# A Traumatic Tick Bite: A Case Report

**DOI:** 10.5811/cpcem.2021.3.50514

**Published:** 2021-04-19

**Authors:** Daniel Finnin, Christopher Hanowitz

**Affiliations:** Albany Medical Center, Department of Emergency Medicine, Albany, New York

**Keywords:** Case report, trauma, anaplasmosis, tick

## Abstract

**Introduction:**

Human granulocytic anaplasmosis is a tick-borne disease with an increasing incidence associated with morbidity and mortality. Uncertainty remains whether a prophylactic dose of doxycycline is effective in prevention.

**Case Report:**

We present a case of an 80-year-old female with syncope, resultant facial trauma, and fever two weeks after a tick bite for which she received prophylaxis. Workup revealed anaplasmosis, and treatment led to symptomatic improvement.

**Conclusion:**

We review the presenting symptoms, laboratory findings, and treatment of anaplasmosis, as well as give caution about the limitations in prescribing a prophylactic dose of doxycycline following a tick bite.

## INTRODUCTION

Human granulocytic anaplasmosis (HGA) is a tick-borne illness caused by the rickettsial bacterium *Anaplasma phagocytophilum* and transmitted by the *Ixodes scapularis* tick (Image).^1^ Symptoms are typically mild resembling viral illness and often self-resolve; however, as many as 3% of victims may have significant morbidity, with up to 1% having meningoencephalitis and 1% mortality.^1^,^3^ The incidence has been increasing since the disease was first recognized in the mid-1990s. In 2018, the most recent year for which data is available, there were 4008 reported cases in the United States. The highest incidence for the disease is in New York, Connecticut, and Wisconsin.^3^,^4^ Coinfection with Lyme disease and babesiosis is common as they are transmitted by the same vector.^5^ Studies in affected areas show a seroprevalence of 8.9–36%.^1^

We present a case of HGA that may have contributed to a syncopal episode leading to traumatic facial injury in a patient who had taken doxycycline prophylaxis for a tick bite.

## CASE REPORT

An 80-year-old female with history of mild chronic obstructive pulmonary disease, mitral valve prolapse, and herniated discs presented to the emergency department (ED) by private vehicle for a facial injury after being struck in the face by a dresser drawer. Leading up to the injury she reported feeling lightheaded, and at the point of losing consciousness she attempted to steady her balance by grabbing a dresser, causing it to fall on her chest pinning her against the wall for approximately five hours. She denied significant pain and noted that the top of her scalp superior to the laceration was insensate. She experienced persistent oozing from the wound and her nose since the injury. A 10-point review of systems was otherwise negative with the exception of fever during the preceding two days to a maximum of 103.3°F on the day of presentation. Notably, she had a tick, which she had removed approximately two weeks prior that was attached for about 24 hours. She was prescribed a one-time dose of 200 milligrams (mg) of doxycycline for Lyme disease prophylaxis, which she had taken on the day of removal by her primary care provider.

On exam, vital signs were notable for heart rate of 102 beats per minute and were otherwise unremarkable. She had a six-centimeter laceration over her left upper forehead with evidence of an open fracture of the frontal sinus on exam with bone fragments visible within the wound. Given the mechanism of injury and her age, we ordered computed tomography of the head, maxillofacial, cervical spine, chest, abdomen, and pelvis, which demonstrated left frontal calvarial fractures. Lab work included the following: a complete blood count; complete metabolic panel; troponin; creatinine kinase; prothrombin time and international normalized ratio; tick panel (Lyme disease immunoglobulin G/immunoglobulin M, *Ehrlichia chaffeensis* polymerase chain reaction [PCR]); *Anaplasma phagocytophilum* PCR; *Babesia microti* PCR; respiratory viral panel; coronavirus disease 2019 PCR; and lactic acid. Notable results are shown in Table 1. An electrocardiogram demonstrated normal sinus rhythm, and the patient was maintained on the cardiac monitor without event. The patient was treated empirically with tetanus toxoid and ampicillin/sulbactam for coverage of the open sinus fracture. Plastic surgery was consulted for evaluation of the open sinus fracture and facial laceration, which they repaired at bedside in the ED. Intravenous fluids were also administered, and trauma surgery was consulted for admission of the patient to their service for further workup and management.

**CPC-EM Capsule**

What do we already know about this clinical entity?

*Human granulocytic anaplasmosis is a tick-borne disease with an increasing incidence associated with morbidity and mortality.*

What makes this presentation of disease reportable?

*We discuss a rare disease with a presentation including a syncopal episode and traumatic injury found to have findings consistent with anaplasmosis.*

What is the major learning point?

*This report highlights the classic historical and laboratory findings of anaplasmosis with caution about limitations of doxycycline in Lyme disease prophylaxis.*

How might this improve emergency medicine practice?

*This case may lead to increased recognition of a rare tick-borne illness to assist with timely diagnosis and treatment.*

During the hospital course, the patient continued to spike fevers and had worsening thrombocytopenia, hyponatremia, and leukopenia as demonstrated in Table 1. To further evaluate for a cardiac etiology of her syncope, an echocardiogram was performed, which did not demonstrate any abnormalities. The *Anaplasma phagocytophilum* PCR ultimately returned positive on hospital day two revealing the diagnosis. She was started on doxycycline 100 mg twice daily with improvement of symptoms and hematologic parameters, and she was subsequently discharged on hospital day six.

## DISCUSSION

Anaplasmosis typically presents with nonspecific symptoms including fever, chills, headaches, and myalgias with associated leukopenia, thrombocytopenia, and elevated transaminases.^6^ The diagnosis should be suspected in a patient with a history of tick bite or tick exposure presenting with these signs and symptoms. Symptoms typically manifest a median of nine days after a tick bite, and patients typically present for healthcare evaluation a few days later.^3^ Diagnosis is confirmed by PCR, buffy coat testing, or serology with a reported sensitivity of 77–80% for both PCR and buffy coat examination.^6^ In our case, the patient was able to relate the history of tick exposure and fever, which helped to guide our workup.

The presentation of HGA as syncope leading to a traumatic event is unusual. There is one report of HGA leading to encephalopathy leading to a motor vehicle crash causing injury.^7^ Another case reported syncope thought to be due to a traumatic splenic rupture caused by HGA requiring resuscitation and surgical intervention.^8^

The specific etiology of syncope in this case is unclear. The suspicion is that HGA could have led to orthostatic hypotension from high fevers leading to increased metabolic demands and dehydration. The patient had an elevated creatinine on presentation that had improved by hospital day two with resuscitation. Human granulocytic anaplasmosis has been previously associated with acute renal failure.^1^ A cardiac etiology is also possible and, notably, the patient had a mild elevation in creatinine kinase and troponin I on presentation, with repeat troponin the next day negative. The troponin elevation could have been from rhabdomyolysis, an arrhythmia that was not identified, non-ST elevation myocardial infarction, blunt cardiac injury, or it could have been direct myocyte injury from HGA, which has been previously described.^9^, ^10^

The treatment of choice is doxycycline, which typically leads to rapid improvement and is continued for 10 days per Infectious Diseases Society of America guidelines. A failure to respond to doxycycline within 48 hours should prompt evaluation for other causes including babesiosis.^6^ Rifampin can be a possible alternative for those with doxycycline intolerance.^10^

There is no data regarding the effectiveness of a single 200-mg dose of doxycycline in preventing non-Lyme tick-borne illness. Doxycycline prophylaxis, a single dose of 200 mg, following a tick bite has been recommended for Lyme disease prevention with criteria as shown in Table 2 with a study demonstrating effectiveness of prevention as 87%. But it is as yet unknown whether this would be helpful for HGA or babesiosis, especially given the preferred treatment with atovaquone and azithromycin in babesiosis.^6^,^10^,^11^ A prophylactic dose of doxycycline was not sufficient to prevent development of HGA in our patient. The emergency clinician should be aware of the limitations of the prophylactic dose and maintain a high index of suspicion for other tickborne illnesses in endemic areas with the appropriate patient history. Further research is needed to determine whether doxycycline prophylaxis following a tick bite is helpful in preventing HGA, but it did not appear to be helpful in this case.

## CONCLUSION

Anaplasmosis is an emerging disease that is increasing in frequency in endemic areas. In the evaluation of fever, hematologic abnormalities, and elevated transaminases with the proper travel history or location, human granulocytic anaplasmosis should certainly be considered as a possible cause. The effectiveness of doxycycline in prophylaxis of HGA has not been evaluated, and the emergency clinician should be aware of its limitations.

## Figures and Tables

**Image:**
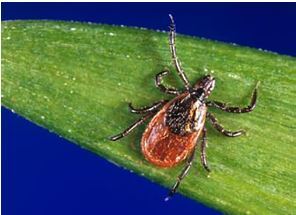
*Ixodes scapularis* tick.^2^

**Table 1:** Lab results for 80-year-old patient with history of tick exposure.

Lab	Facility lab range	Admission	Hospital day two
Sodium	135–145 mEq/L	134 mEq/L	133 mEq/L
Potassium	3.4–5.2 mEq/L	4.1 mEq/L	3.3 mEq/L
Chloride	99–109 mEq/L	100 mEq/L	103 mEq/L
Bicarbonate	21–30 mEq/L	24 mEq/L	22 mEq/L
Blood urea nitrogen	7–22 mg/dL	24 mg/dL	13 mg/dL
Creatinine	0.6–1.2 mg/dL	1.77 mg/dL	0.95 mg/dL
Glucose	65–99 mg/dL	124 mg/dL	135 mg/dL
Aspartate transaminase	5–45 IU/L	99 IU/L	
Alanine transaminase	5–60 IU/L	60 IU/L	
Creatine kinase	30–225 IU/L	1495 IU/L	
Troponin I	0–0.04 ng/mL	0.05 ng/mL	0.03 ng/mL
Hemoglobin	11.0–14.7 g/dL	12.1 g/dL	10.0 g/dL
Hematocrit	33.0–44.0%	36.3%	30.6%
White blood cells	4.1–9.3 10^3^/uL	3.7 10^3^/uL	1.7 10^3^/uL
Platelets	130–350 10^3^/uL	81 10^3^/uL	40 10^3^/uL

*mEq*, milliequivalent; *L*, liter; *mg*, milligrams; *dL*, deciliter; *IU*, international units; *ng*, nanograms; *mL*, milliliter; *g*, grams; *uL*, microliter.

**Table 2:** Criteria for doxycycline prophylaxis after tick bite.

The tick is identified as an adult or nymph *Ixodes scapularis.*
Prophylaxis is started within 72 hours of removing tick.
Local rate of infection is ≥20%.
Doxycycline is not contraindicated.
The tick has been attached for ≥36 hours based on time of exposure or engorgement.
